# RETRACTED: Sathish et al. Influence of Compression Molding Process Parameters in Mechanical and Tribological Behavior of Hybrid Polymer Matrix Composites. *Polymers* 2021, *13*, 4195

**DOI:** 10.3390/polym18141684

**Published:** 2026-07-08

**Authors:** Thanikodi Sathish, Vinayagam Mohanavel, Thandavamoorthy Raja, Sinouvassane Djearamane, Palanivel Velmurugan, Omaima Nasif, Saleh Alfarraj, Ling Shing Wong, Velu Manikandan, Manikkam Ravichandran

**Affiliations:** 1 Department of Mechanical Engineering, Saveetha School of Engineering, Saveetha Institute of Medical and Technical Sciences, Chennai 602105, India; 2 Centre for Materials Engineering and Regenerative Medicine, Bharath Institute of Higher Education and Research, Chennai 600073, India; mohanavel2k16@gmail.com (V.M.); palanivelvelmurugan@bharathuniv.ac.in (P.V.); 3 Department of Mechanical Engineering, Vel Tech Rangarajan Dr. Sakunthala R&D Institute of Science and Technology, Chennai 600062, India; rajasd28@gmail.com; 4 Faculty of Science, Universiti Tunku Abdul Rahman, Kampar 31900, Malaysia; 5 Department of Physiology, College of Medicine and King Khalid University Hospital, King Saud University, Medical City, P.O. Box 2925, Riyadh 11461, Saudi Arabia; onasif@ksu.edu.sa; 6 Zoology Department, College of Science, King Saud University, Riyadh 11451, Saudi Arabia; salfarraj@hotmail.com; 7 Faculty of Health and Life Sciences, INTI International University, Nilai 71800, Malaysia; lingshing.wong@newinti.edu.my; 8 Division of Biotechnology, College of Environmental & Bioresource Sciences, Jeonbuk National University, Iksan 570752, Republic of Korea; mani.env2014@gmail.com; 9 Department of Mechanical Engineering, K. Ramakrishnan College of Engineering, Trichy 621112, India; smravichandran@hotmail.com

The Journal retracts the article “Influence of Compression Molding Process Parameters in Mechanical and Tribological Behavior of Hybrid Polymer Matrix Composites” [1] cited above.

Following publication, concerns were brought to the attention of the Editorial Office regarding the reliability of a figure presented in this article [1].

In accordance with standard journal procedures, the Editorial Office and Editorial Board conducted an investigation that confirmed indications of inappropriate editing in Figure 15. Additionally, the investigation identified an image duplication within Figure 2. Although the authors cooperated with the investigation, the explanations and supporting materials provided were not deemed sufficient by the Editorial Board to resolve the identified concerns. Consequently, the Editorial Board has lost confidence in the reliability of the findings and decided to retract this publication [1], as per MDPI’s retraction policy.

This retraction was approved by the Editor-in-Chief of the journal *Polymers*.

The authors have been informed of this retraction and have indicated their agreement with this decision.
